# Supplementary material for: 1H Nuclear Magnetic Resonance (NMR)-Based Cerebrospinal Fluid and Plasma Metabolomic Analysis in Type 2 Diabetic Patients and Risk Prediction for Diabetic Microangiopathy
Source: J Clin Med. 2019 Jun 19;8(6):874. doi: 10.3390/jcm8060874 (PMC6616639; doi:10.3390/jcm8060874)
Supplement: Supplementary file 1 [file jcm-08-00874-s001.pdf]

Supplementary Materials:

Table S1. Association of altered metabolites in CSF and plasma samples with the presence of T2DM.

Comparison	T2DM vs. control			
**Significantly changed metabolites in CSF**	**Crude OR**	***p***	**Adjusted OR [a]**	***p***
**CSF glucose**	1.062	<0.001*	1.061	0.002*
Alanine	1.009	<0.001*	1.009	0.002*
Glycine	1.023	<0.001*	1.001	0.001*
Histidine	0.906	<0.001*	0.871	0.001*
Mannose	1.035	<0.001*	1.040	0.002*
2-hydroxybutyrate	1.005	0.003*	1.004	0.036*
Lactate	1.000	<0.001*	1.001	0.001*
Tyrosine	1.021	0.002*	1.029	0.003*
Leucine	1.000	<.0001*	1.039	0.004*
Phenylalanine	1.000	0.007*	1.033	0.033*
Pyruvate	1.000	0.006*	1.003	0.049*
Valine	1.012	0.003*	1.008	0.037*
Alanine, Glycine, Histidine, Mannose, Pyruvate	2.618	0.003*	4.364	0.005*
**Significantly changed metabolites in plasma**	**Crude OR**	***p***	**Adjusted OR [a]**	***p***
Plasma glucose	1.070	<0.001*	1.092	0.003*
Plasma HbA1c	1.207	0.002*	1.288	0.019*
Plasma HOMA-IR	1.732	0.002*	1.712	0.027*
Alanine	0.999	0.015*	0.998	0.004*
Citrate	0.991	0.002*	0.988	0.003*
Histidine	0.977	0.002*	0.974	0.003*
Lipid (CH2-CH=CH)	1.000	0.005*	1.000	0.043*
N-acetyl glycoprotein	1.000	<0.001*	1.000	0.003*
Phenylalanine	1.024	<0.001*	1.024	0.001*
Glutamine	1.000	0.001*	0.997	0.001*
Acetate	1.004	0.099	1.006	0.028*
Leucine	1.000	0.002*	1.004	0.036*
Acetate, Citrate, Histidine, Leucine, N-acetyl-glycoprotein	2.718	<.0001*	5.355	<0.001*

Abbreviation: T2DM, type 2 diabetes mellitus; OR, odds ratio; HbA1c, glycated hemoglobin A1c; HOMA-IR, Homeostatic Model Assessment for Insulin Resistance [a] Adjusted for age, sex, body mass index (BMI), and medications for chronic diseases (hypertension, hyperlipidemia) * $p < 0.05$.

(A) CSF

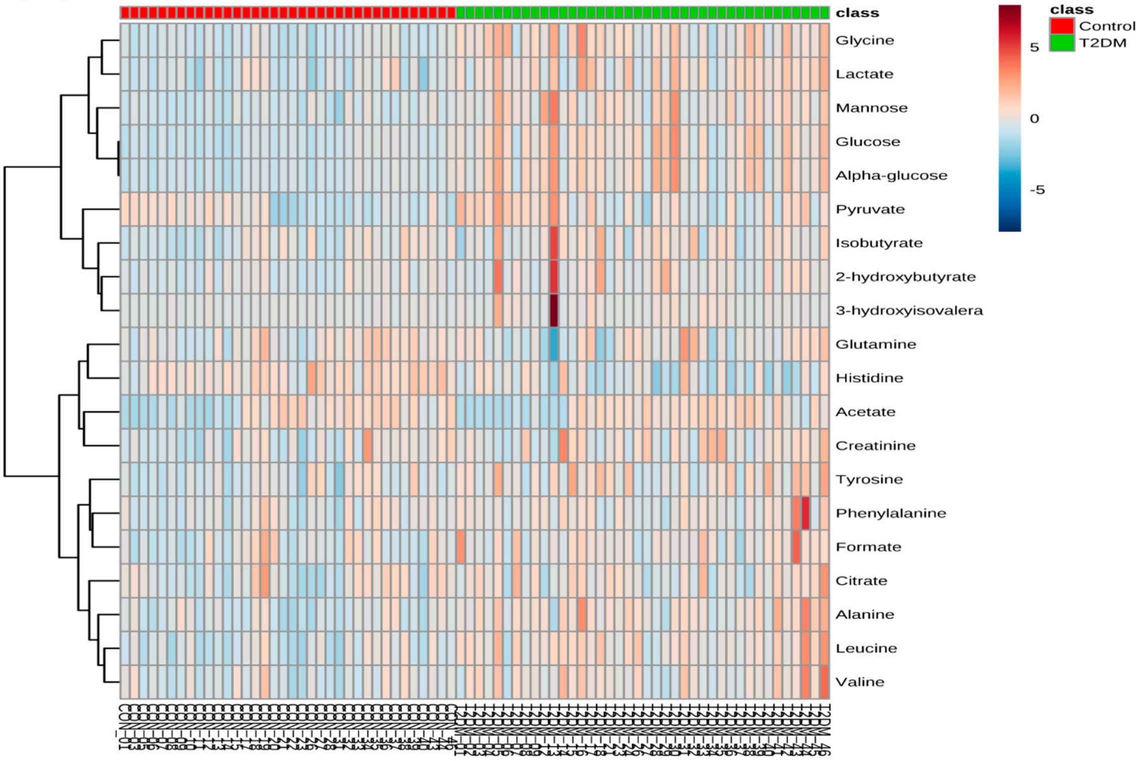

(B) Plasma

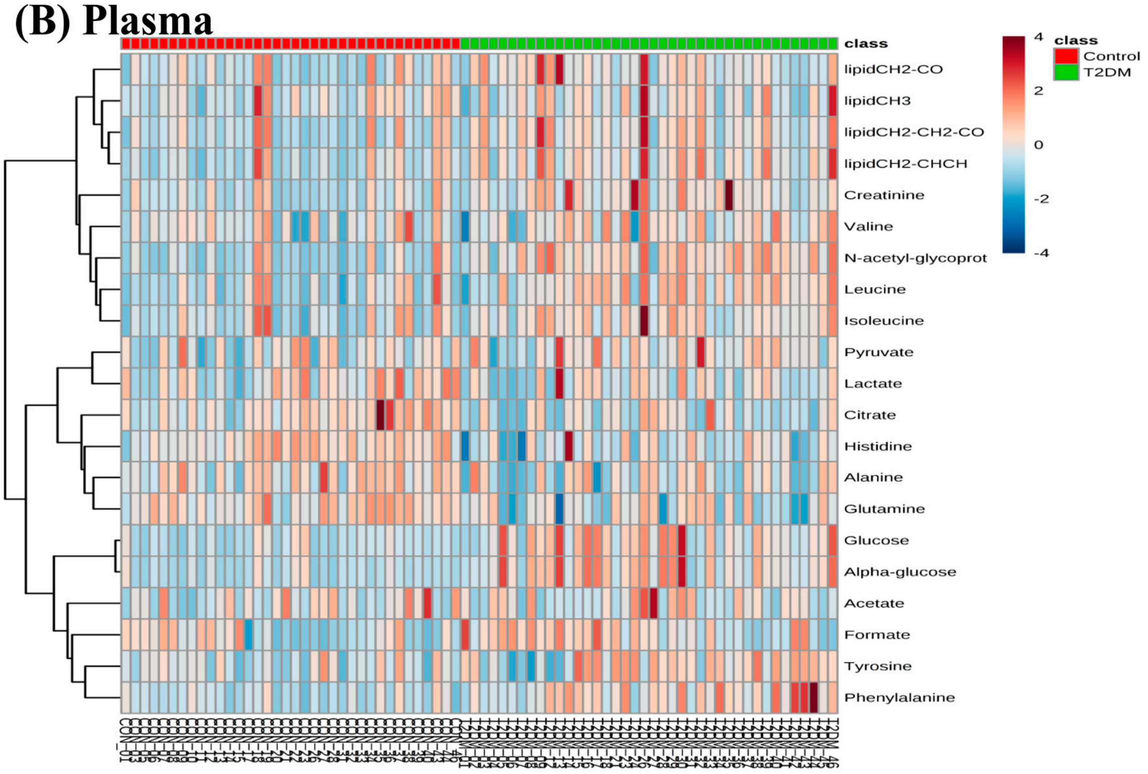

Figure S1. Metabolite heatmaps in (**A**) CSF and (**B**) plasma samples from T2DM patients and control subjects.

(A) CSF

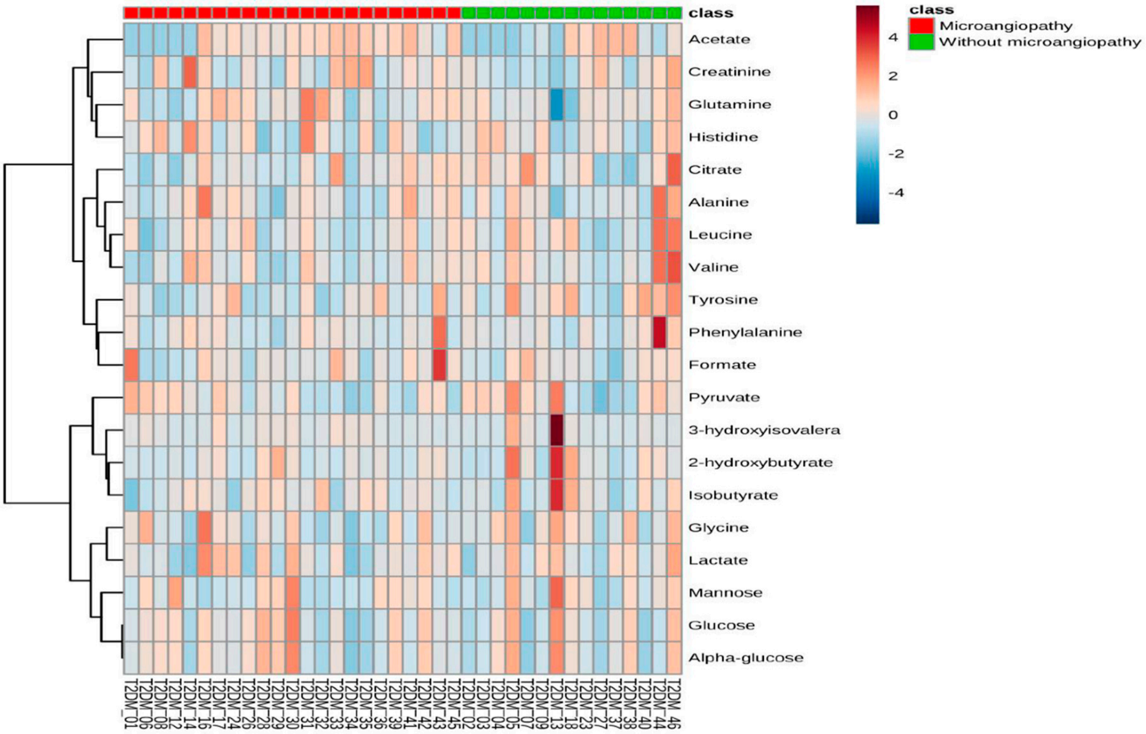

(B) Plasma

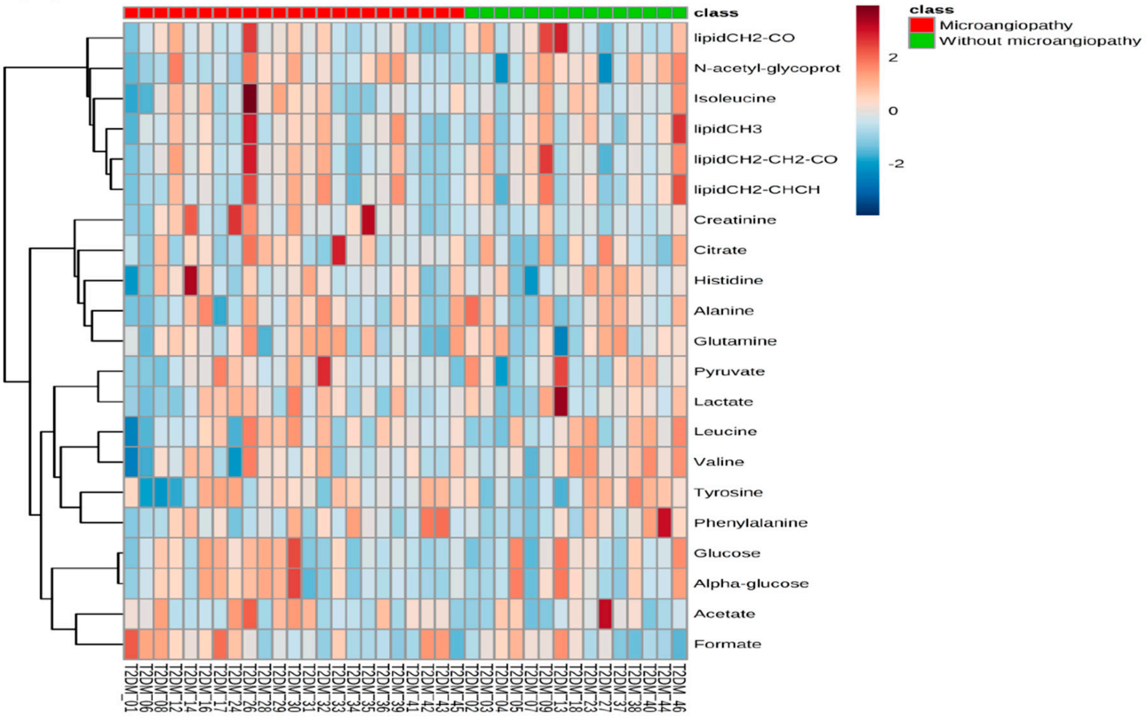

Figure S2. Metabolite heatmaps in (**A**) CSF and (**B**) plasma samples from T2DM patients with microangiopathy versus T2DM patients without microangiopathy.

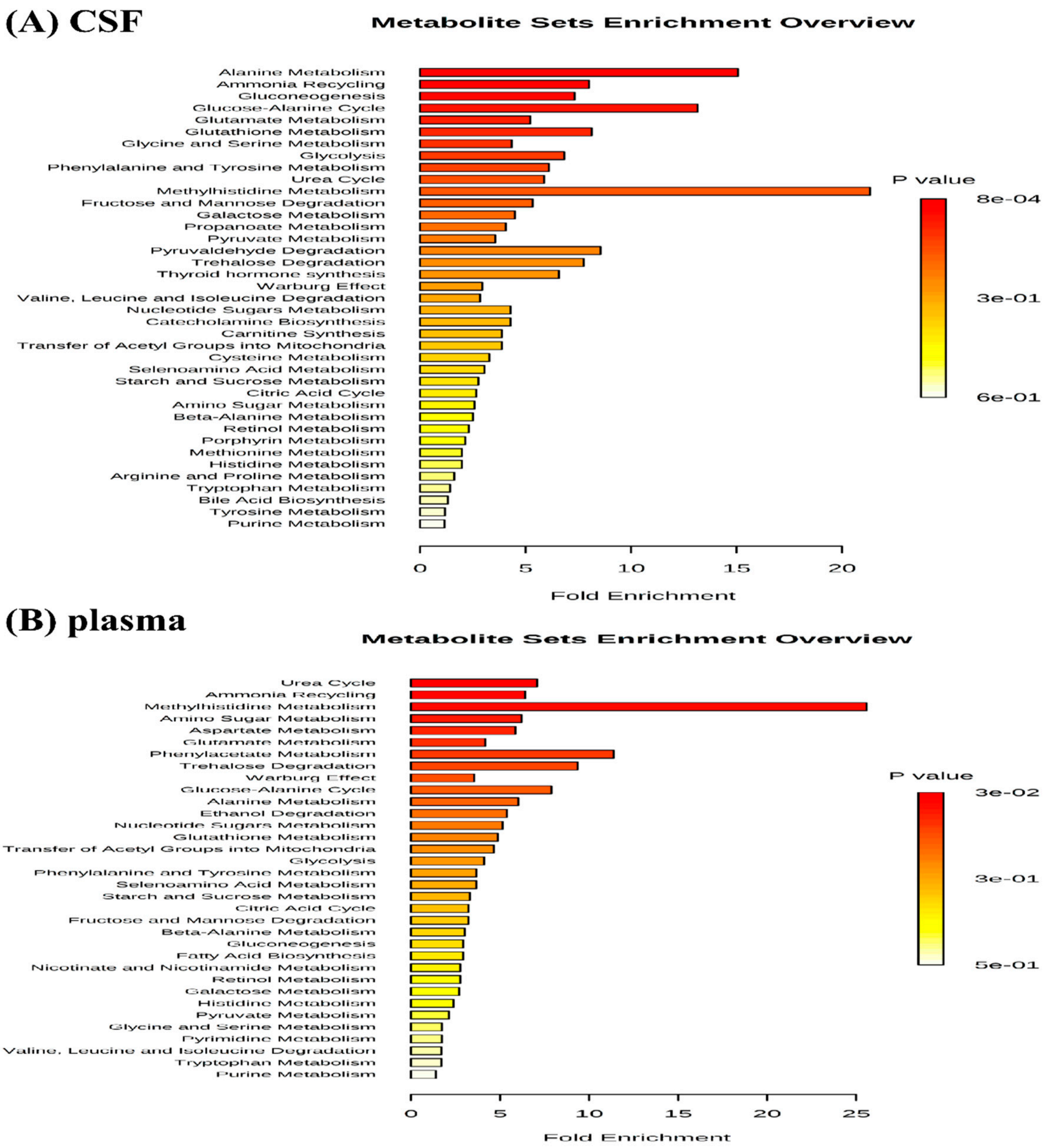

Figure S3. Enrichment analysis of involved metabolic pathways for significant metabolites in (**A**) CSF (**B**) plasma samples from T2DM patients.
